# Magnetic Resonance Spectroscopy of Hepatic Fat from Fundamental to Clinical Applications

**DOI:** 10.3390/diagnostics11050842

**Published:** 2021-05-07

**Authors:** Duanghathai Pasanta, Khin Thandar Htun, Jie Pan, Montree Tungjai, Siriprapa Kaewjaeng, Hongjoo Kim, Jakrapong Kaewkhao, Suchart Kothan

**Affiliations:** 1 Center of Radiation Research and Medical Imaging, Department of Radiologic Technology, Faculty of Associated Medical Sciences, Chiang Mai University, Chiang Mai 50200, Thailand; duanghathai.pa@gmail.com (D.P.); ktdhtun28@gmail.com (K.T.H.); jiepan@sdnu.edu.cn (J.P.); mtungjai@gmail.com (M.T.); siriprapa.k@cmu.ac.th (S.K.); 2 Shandong Provincial Key Laboratory of Animal Resistant Biology, College of Life Sciences, Shandong Normal University, Jinan 250014, China; 3 Department of Physics, Kyungpook National University, Daegu 41566, Korea; hongjoo@knu.ac.kr; 4 Center of Excellence in Glass Technology and Materials Science (CEGM), Nakhon Pathom Rajabhat University, Nakhon Pathom 73000, Thailand; jakrapong@webmail.npru.ac.th

**Keywords:** magnetic resonance spectroscopy, liver fat, hepatic steatosis, liver fat fraction, 1H-MRS

## Abstract

The number of individuals suffering from fatty liver is increasing worldwide, leading to interest in the noninvasive study of liver fat. Magnetic resonance spectroscopy (MRS) is a powerful tool that allows direct quantification of metabolites in tissue or areas of interest. MRS has been applied in both research and clinical studies to assess liver fat noninvasively in vivo. MRS has also demonstrated excellent performance in liver fat assessment with high sensitivity and specificity compared to biopsy and other imaging modalities. Because of these qualities, MRS has been generally accepted as the reference standard for the noninvasive measurement of liver steatosis. MRS is an evolving technique with high potential as a diagnostic tool in the clinical setting. This review aims to provide a brief overview of the MRS principle for liver fat assessment and its application, and to summarize the current state of MRS study in comparison to other techniques.

## 1. Introduction

Fatty liver, caused by the accumulation of excess fat in the liver, is a common characteristic of liver diseases such as alcoholic fatty liver disease (AFLD) and nonalcoholic fatty liver disease (NAFLD). A recent study showed that NAFLD is the most common form of hepatic steatosis, with a high prevalence, of approximately 13–32% in the general population, that is higher in Western countries [1]. The prevalence of NAFLD is especially high in people with metabolic syndrome or diabetes [2]. Several lines of evidence also indicate a positive relationship between obesity and NAFLD. The increasing prevalence of NAFLD in children, young adults, and adults is a growing public health concern worldwide [3,4]. Moreover, the association between NAFLD and liver-related morbidity and mortality has led to growing interest in hepatic steatosis evaluation. An increasing number of NAFLD cases worldwide has led to an increasing interest in noninvasive techniques to accurately investigate liver fat content in vivo [5]. Previous studies showed that donors with mild steatosis could potentially have an increased morbidity risk in living-donor transplants and that livers from these donors could lead to liver transplant failure [6]. Therefore, this suggests the crucial role of accurate liver fat evaluation in the clinical setting.

Several imaging modalities have been used in liver fat assessment, including ultrasound (US), computed tomography (CT), magnetic resonance imaging (MRI), and magnetic resonance spectroscopy (MRS). Among these imaging modalities, MRS has shown high accuracy in liver fat quantification with safe, noninvasive, and reproducible results [7]. MRS provides a direct measurement of liver fat from the signal peak of fat and is also commonly accepted as a noninvasive reference standard for liver fat assessment [8,9].

The aim of this review is to provide a brief overview of the MRS technique from its fundamental aspects to its successes in comparison to other available liver fat quantification techniques. Additionally, we aim to provide examples of MRS’s role in key areas in both research and clinical practice.

## 2. Why Is a Sensitive Method for Liver Fat Assessment Important?

Hepatic steatosis or the accumulation of liver fat is the pathological hallmark of NAFLD and has many other clinical implications. It is estimated that up to 30% of NAFLD patients may have NASH and have a greater risk of progression to end-stage liver disease [10]. Notably, NAFLD is the second most common etiology for liver transplantation and is projected to become the most common indication within the next decade [11,12,13]. Several studies have shown that increased liver fat content is also associated with non-liver-related mortality and morbidity. Liver steatosis was suggested to be an independent risk factor for cardiovascular disease [14], kidney disease [13], and cancer [15]. NAFLD patients have a 34 to 69% higher chance of dying over the next 15 years than individuals in the general population [16].

The criterion for the diagnosis of NAFLD is excess fat accumulation in the liver that affects more than 5% of hepatocytes [17]. The buildup of fat within the liver can impair hepatocyte function, which can progress to hepatic inflammation, fibrosis, cirrhosis, and even hepatocarcinoma [18]. NAFLD comprises a wide spectrum of diseases, ranging from simple steatosis to the more aggressive form of NAFLD with hepatocyte injury and inflammation categorized as nonalcoholic steatohepatitis (NASH) [19]. Patients with NASH face a higher risk of cardiovascular disease, liver failure, cirrhosis, and liver cancer than those with simple steatosis [17]. One of the most common steatosis grading systems is the NASH Clinical Research Network (NASH CRN-NAS), which is based on the histological evaluation from a liver biopsy. The degree of liver steatosis is stratified by the percentage of hepatocytes affected by steatosis, as follows: S0 (<5%), S1 (5–33%), S2 (>33–66%), and S3 (>66%) [20].

Currently, the gold standard for identifying more aggressive NASH is a liver biopsy in staging fibrosis and liver steatosis [1]. In addition to providing information for staging liver steatosis, a liver biopsy also assists with identification of the manifestation of other liver diseases that might coexist or have similar characteristics to fatty liver, such as chronic hepatitis C infection [21]. However, the percutaneous liver biopsy is limited by its invasive nature and is not suitable for clinical applications that require real-time monitoring of liver fat levels throughout therapeutic intervention. Moreover, liver biopsy samples have only a small volume of liver parenchyma and show both inter- and intraobserver variability [22]. While fat accumulates within the liver in a diffuse pattern, the distribution of fat in the liver parenchyma is heterogeneous [23,24]. Therefore, a liver biopsy may not be an accurate representation of liver health. 

Notably, an early stage of NAFLD can easily be reversed with lifestyle modification [25]. While the identification of this early stage may help prevent disease progression, a sensitive and noninvasive method for liver steatosis will prove to be more useful in later stages of NAFLD. Considering the prevalence and severe consequences of advanced NAFLD, sensitive and real-time monitoring tools would help with the evaluation of the therapeutic response that might lead to small changes in liver fat in the early stage of intervention. At present, serum biomarkers and imaging techniques have been proposed as two main approaches for noninvasive liver steatosis assessment [26], and MRS is one of those techniques.

## 3. Available Imaging Modalities for Liver Fat Assessment Compared to MRS

Conventional US is inexpensive and readily available in many clinical settings for the diagnosis of fatty liver. However, US is highly operator dependent and reported to have low accuracy and reproducibility [17]. US is also not specific for liver fat. US estimates the liver fat content through the attenuation of soundwaves, which are also attenuated by many other liver diseases, such as fibrosis, hepatitis, and hemochromatosis [11,27,28]. The method is also potentially difficult to perform in individuals with high body mass index (BMI) and those at high risk for liver fat accumulation [29,30]. Additionally, US is not sensitive to mild steatosis and exhibits a small alteration in liver fat level with a sensitivity of only 60.9 to 65% [31,32].

Transient elastography (TE) is an ultrasound-based modality that simultaneously measures liver steatosis and fibrosis. TE transducer produces a low-frequency (50 Hz) shear wave that propagates through the liver tissue, followed by a pulse-echo ultrasound that measures its velocity, which reflects the degree of liver stiffness or liver stiffness measurement (LSM) [33,34,35,36]. The controlled attenuation parameter (CAP) estimates the degree of ultrasound attenuation by hepatic fat at the central frequency of transient elastography [37]. CAP is expressed in decibels per meters (dB/m), with typical CAP ranges from 100 to 400 dB/m [38], with the higher CAP value reflecting higher liver fat content. CAP value is machine independent since it uses the standardized (controlled) setting. While CAP is noninvasive and cost-effective, CAP reported high measurement failure rates in obese individuals [39,40]. The reliable criteria for CAP are still a topic of ongoing debate [37,41]. Although applying XL probes potentially solve the drawback of CAP in high BMI individuals [42], it may not be reliable in severely obese individuals due to the lack of reference criteria [40].

Computed tomography can provide an objective assessment of hepatic steatosis from the measurement of radiation attenuation value. Fatty liver has a lower attenuation value than normal liver parenchyma. Unenhanced CT has demonstrated higher accuracy than enhanced CT due to the increased liver attenuation from CT contrast agents [43]. In addition, single-energy CT also shows a high correlation with MRS (r^2^ = 0.86) and is reported to be more accurate than dual-energy CT (r^2^ = 0.423) [44]. However, unenhanced CT still has a limited sensitivity of 50% and specificity of 77.2% for mild steatosis, and a sensitivity of 72.7% and specificity of 91.3% for moderate-to-severe steatosis [11,45]. Another limitation is that the presence of fibrosis, edema, or iron deposition within the liver potentially affects the reliability of steatosis assessment [46]. While unenhanced CT provides a more quantitative assessment than US or TE, it has lower accuracy than MRI and MRS [11]. Moreover, the utilization of ionizing radiation in CT therefore renders this method unsuitable for repeated measurements in sensitive populations, such as children or pregnant women.

Various MRI techniques have been developed for liver fat assessment, including conventional in-phase (IP) and opposed-phase (OP) imaging, fat-suppressed imaging, and chemical shift imaging (CSI). MRI uses the magnetic properties of protons under a magnetic field to generate the signal for image formation. This signal contains information from all kinds of chemical compositions, including fat and water, the origin of most signals for MR imaging. MRI exploits the properties of precession frequency differences or between water and fat for liver fat assessment. Protons within water (W) precess faster than those in fat (F) and alternate between in-phase (IP; W+F) and opposed-phase (OP; W-F) at predictable intervals [29]. This difference in resonance frequency between two protons in a static magnetic field also refers to a chemical shift. The relative chemical shift between water and fat is approximately 3.5 parts per million (ppm) [47]. The relative signal difference between IP and OP images could then be used for the subjective assessment of liver fat [11]. However, this method requires the correction of multiple confounding issues, especially for mild steatosis, and is limited by the upper limit for liver steatosis at 50% [48]. These confounding issues in conventional MRI techniques include T1 bias, T2* decay effect, spectral complexity, and iron deposition [29].

CSI also exploits the chemical shift property to fully separate the fat and water signal into fat- or water-only images. This technique potentially allows fat quantification from mild steatosis up to 100%. However, CSI also requires correction for the same confounding issues as IP and OP imaging. In addition, CSI does not directly measure fat and water concentrations within the liver [22]. While the CSI technique demonstrates high accuracy and ease of application in the clinical setting, it does not provide direct measurement from fat signals within the liver like the MRS technique does. Compared to CSI, MRS provides more accurate and direct noninvasive measurements of liver fat. Several studies have regarded MRS as a standard method for validation during the development of new techniques, including CSI [49,50].

There are also other available options for noninvasive quantification of liver fat, for example, MRI-estimated proton density fat fraction (MRI-PDFF) and Multi Echo Dixon. 

While MRI-PDFF allows mapping of the whole liver, it require priori knowledge of the multi-peak fat spectral model to accurately measure triglyceride composition [51,52]. Additionally, the MRI-PDFF technique does not directly measure the fat signal and its reported performance in grading of hepatic steatosis was inconsistent [7,53]. The other technique, the 3D Multi-Echo Dixon, can be used to evaluate both liver steatosis and iron deposition at the same time [54]. Although promising, the relaxation rate R2* (1/T2*) may be affected by fibrosis without iron overload and reported a failure rate of from 4 to 14% due to fat–water swap [55,56,57], while MRS reported a lower failure rate [57]. For more information on MRI techniques beyond the scope of this review, please refer to more detailed review articles [18,29,58,59].

Among the available options for liver fat assessment, the MRS technique is considered to be the most accurate and effective method, providing quantitative concentrations with high sensitivity to subtle changes in liver fat [60].

MRS is superior to other noninvasive methods, in particular due to the fact that MRS-derived liver fat fraction is not affected by iron deposition, fibrosis, or coexisting liver pathology and allows absolute quantitative measurements of liver fat. Moreover, MRS has demonstrated excellent performance for the detection and grading of liver steatosis. MRS was previously reported to have high sensitivity and specificity of 94.4% and 89.5%, respectively [61,62], which is higher than both US and CT. MRS has also demonstrated superior performance in detection and grading compared to controlled attenuation parameters from elastography [63]. Table 1 compares the characteristics, advantages, and disadvantages of commonly used imaging modalities for liver fat assessment. 

The next sections will focus on providing a brief overview of the principle of the MRS technique and discuss common limitations and its applications. For more information on MRS techniques beyond the basics about MRS, please refer to more detailed review articles and publications, for example [64,65,66].

**Table 1:** Imaging techniques for liver fat assessment.

Method	Assessment for Liver Fat	Advantages	Disadvantages	Possible Confounders
US	Nonquantitative Mild steatosis: Sensitivity 55.3–66.6%, Specificity 77.0–93.1% [22,45,61] Moderate-to-severe steatosis: Sensitivity 79.7–90%, Specificity 86.2–95% [61,67,68]	Noninvasive Readily available in clinical setting Relatively inexpensive	Nonqualitative Indirect measurement Low accuracy for mild steatosis and steatosis grading Modest diagnostic accuracy User dependence	Iron deposition, fibrosis, edema, hepatitis, ascites, and obesity [31,32]
CAP	Relative Quantitative Mild steatosis: Sensitivity 87%, Specificity 91%. Moderate steatosis: Sensitivity 85%, Specificity 74%. Severe steatosis: Sensitivity 76%, Specificity 58% [40]	Noninvasive Ease of measurement Operator-independence Relatively inexpensive	Required further validation Low accuracy in severe steatosis	Acute hepatitis, chronic hepatitis, ascites. Narrow intercostal space, high visceral fat, obesity [37,69]
CT	Relative Quantitative Mild steatosis: Sensitivity 50%, Specificity 77.2% [45] Moderate-to-severe steatosis: Sensitivity 72.7%, Specificity 91.3% [45]	Readily available in clinical setting Easy to perform Simple to analyze	Uses ionizing radiation Indirect measurement Low accuracy for mild steatosis	Iron deposition, edema, glycogen, and amiodarone Unenhanced CT is preferred [43,46]
MRI	Relative Quantitative All degrees of steatosis: IP and OP method; Sensitivity 82–90%, Specificity 89.9–91% [8]	Noninvasive Can be used in sensitive groups. Possible detectability 0–100% dynamic range after correction for confounders Allows liver fat mapping of the entire liver	Relatively expensive Indirect measurement of liver fat but from the assessment of signal loss during IP and OP echoes. Requires correction for confounding factors	Iron deposition, fibrosis, and severe steatosis Contraindications for MRI scanner [48]
MRS	Relative Quantitative All degrees of steatosis: Sensitivity = 94.4%, specificity = 89.5% [61,62]	Directly measures a signal from liver fat. Allows absolute quantitative measurement. Not affected by iron deposition, fibrosis, or coexisting liver pathology	Relatively expensive Usually samples only small area of liver Analysis methods are complex and require user expertise Requires correction for confounding factors for accurate quantification	Variability between MR vendors, pulses sequence, and method of analysis Contraindications for MRI scanner

US: Ultrasound; CT: Computed Tomography; MRI: Magnetic Resonance Imaging; MRS: Magnetic Resonance Spectroscopy; CAP: Controlled Attenuation Parameter.

## 4. Basic Principle for MRS

**Figure 1:**
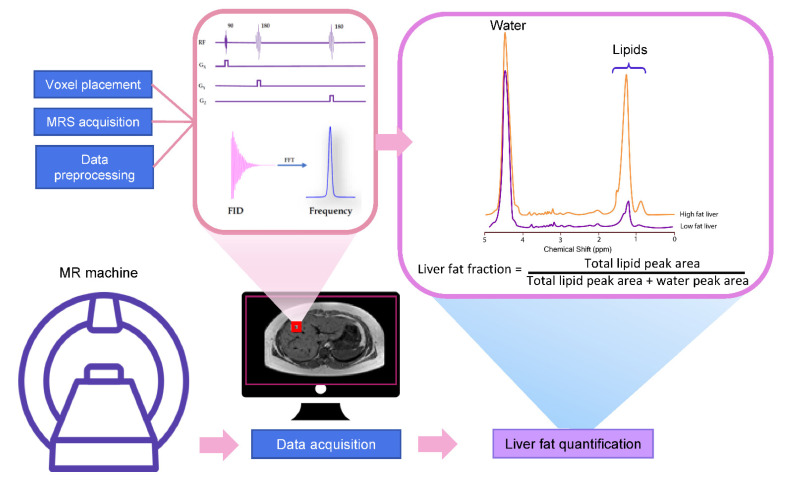
Schematic explanation of how to acquire the MR spectrum. MR spectra were obtained from a region of interest. Free induction decay (FID) is then acquired and converted to resonance frequency spectrum by fast Fourier transformation (FFT). The liver fat fraction can be calculated from the peak area corresponding to fat and water.

MRS utilizes the MR principle to identify and quantify the metabolite from the tissue of interest. The signal in MRS is obtained in the same way as MR imaging, that is, a radiofrequency (RF) at specific resonance is applied to nuclei (e.g., ^1^H, ^13^C, ^31^P, etc.) in a static magnetic field to generate a signal [70]. The pulse sequence and MR signal acquisition are shown in Figure 1. This signal comes from a specific area of interest or the voxel that is then Fourier transformed from the MR signal to the MR spectrum. Unique chemical properties and environments lead to the unique proton resonance frequency and peak shape of each metabolite. This slight shift of the resonance position along the x-axis of the spectrum is termed the chemical shift, which is measured in ppm [64]. The calculation of ppm is obtained from the distance in Hertz (Hz) relative to a reference peak such as Si(CH_3_)_4_ or water, divided by the operating frequency of the MR system [64,71]. The proton resonance frequency is proportional to the MR field strength at 63.9 MHz at 1.5 Tesla, 127.8 MHz at 3 Tesla, and 298.2 MHz at 7 Tesla. Therefore, the chemical shift in ppm can be compared across studies irrespective of MR field strength. The MR field strength is also proportional to the improved signal-to-noise ratio (SNR). Thus, the increased field strength of MR machines improves spectral resolution and the separation of metabolite peaks [72].

### 4.1. Liver MRS Spectrum

**Figure 2:**
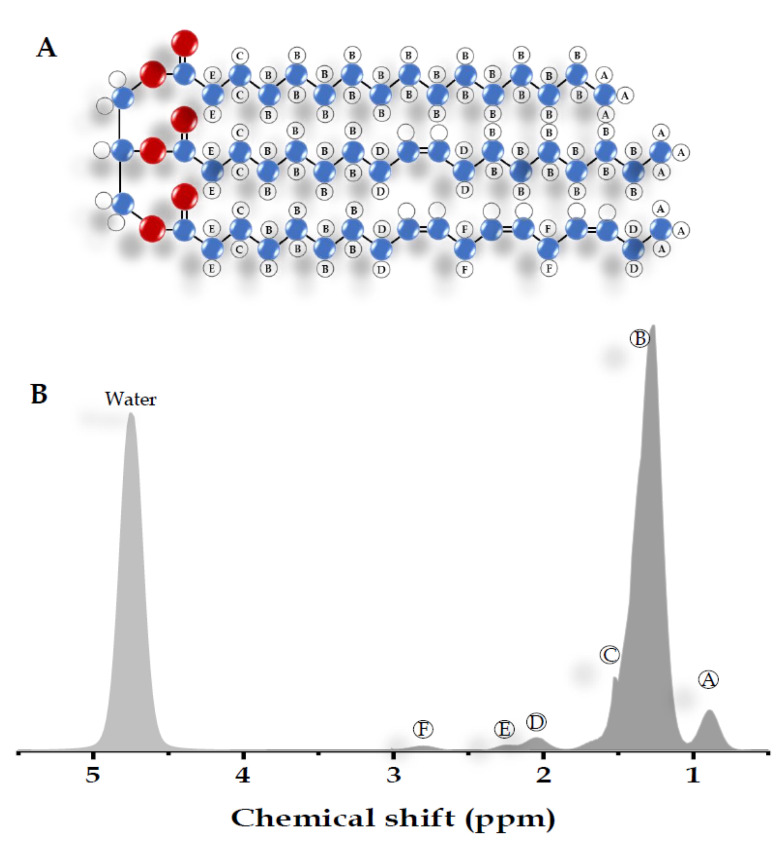
Schematic illustration of a triglyceride molecule and MR spectrum from the liver. (**A**) The molecular structure of the triglyceride. Hydrogen atoms are shown in white (○), carbon in blue (●), and oxygen in red (●). (**B**) The spectrum of all molecules obtained from the liver corresponding to the hydrogen atom position on the molecular structure of a triglyceride.

Most of the visible peaks in the MRS liver spectrum obtained from the clinical MR scanner (1.5-3 Tesla) are fat and water. While water shows a single peak at approximately 4.7 ppm, fat shows multiple peaks due to its complex chemical components (Table 2) [22,73,74]. Six resonances of fat are usually detected with the main lipid peak at approximately 0.9 to 2.75 ppm. There are also unresolved fat resonances at 4.2 and 5.3 ppm from glycerol and olefinic acid, respectively (Figure 2) [73,74,75]. These two peaks overlap with the water peak signal at 4.7 ppm. While the correct identification of the liver fat peak is possible in MR systems with a high field, it is less feasible in a lower field with lower spectral resolution and broader linewidth. The misidentification of lipid peaks leads to quantification errors in liver fat; therefore, these unresolvable peaks are not qualified for diagnostic purposes [64].

Chemical shifts of each peak were achieved at different hydrogen atom positions from the triglyceride molecule on the human liver by a 1.5 Tesla MRI machine.

### 4.2. The Acquisition of Liver MRS Spectrum

MRS liver spectra are often obtained using a single-voxel technique [18,22]. The advantage of the single-voxel technique is that it provides a high SNR from a large volume of liver sampled. While multivoxel spectroscopy allows larger coverage of the liver than other techniques, the distance from the coil to the organ, longer acquisition time, and reduced shim quality limit its application [22,71,76].

Typically, single-voxel MRS is usually performed with a voxel size of 2 × 2 × 2 cm^3^ or 3 × 3 × 3 cm^3^ [18]. A large voxel size might be preferable since it provides more SNR and thus reliable liver fat quantification with a shorter acquisition time [71,77]. The voxel is manually placed in the liver parenchyma using multiplanar MR images and avoids large vessels, bile ducts, and edges of the liver. The voxel edge should be positioned more than 10 mm from the inner margin from the abdominal wall to avoid contamination of the subcutaneous fat signal [21,60]. A coil with a multichannel coil array receiver is recommended over a body coil for MRS acquisition to maximize the SNR [64,78]. The quality of the MRS spectrum is sensitive to inhomogeneous magnetic fields. Good magnetic field homogeneity is required for good spectral resolution or small line width to distinguish peaks from each other. The use of shimming of the magnetic field is therefore necessary to minimize field inhomogeneity across the voxel. While most commercially available MR machines have automated shimming prior to MRS acquisition, manual shimming can also be performed to improve field homogeneity.

The most common pulse sequences for MRS spectral acquisition are stimulated-echo acquisition mode (STEAM) and point-resolved spectroscopy (PRESS). STEAM is a stimulated echo-based technique that utilizes three 90° angles to create well-defined voxels and reduce contaminating signals outside the voxel [18]. PRESS is a spin echo-based technique that uses a 90° pulse followed by 180°–180° (Figure 1). While PRESS provides a double SNR compared to STEAM, it is more affected by J-coupling and overestimated fat fraction [79,80]. Therefore, STEAM may be a preferable choice for accurate liver fat quantification.

MRS spectra should be obtained without water and fat suppression since both signals are required for the calculation of the fat fraction ratio. Additionally, the spatial saturation band should not be employed since it potentially partially saturates the fat and water signals, causing errors in the fat fraction calculation [18,76].

### 4.3. MRS Spectrum Analysis and Liver Fat Quantification

Several commercial and noncommercial software programs are available for the analysis of the MRS spectrum [81,82,83,84]. These specialized software packages provide more flexibility for MRS spectra analysis from the preprocessing process through metabolite quantification. The additional details on software packages for liver fat quantification is available in Appendix A.

The liver fat fraction calculation can be obtained from the ratio of lipid peak area to the water peak area. After the visible lipid peaks (0.9–2.1 ppm) and water (4.7 ppm) are identified in the spectrum analysis process, the area under the peak is calculated through peak modeling, such as the Gaussian or Lorentzian model [76]. As previously discussed, only disguisable lipid peaks (0.9, 1.3, and 2.1 ppm) or main lipid peaks (1.3 ppm) have been used for the calculation of lipid signals [21]. A total fat signal is obtained from the summation of individual lipid peak areas from water-suppressed liver MRS spectra. The total water signal is obtained from unsuppressed spectra. The fat fraction (FF) can then be calculated by dividing the total fat signal by the sum of the water and fat signals (FF = Signal_fat_/Signal_fat_ + Signal_water_) [85].

MRS-derived FF is generally accepted as a reference method for noninvasive liver fat assessment [44,86,87,88]. MRS-derived FF is used as a standard reference in the validation of the method for proton-density FF from MR imaging [86] and other imaging modalities, such as CT and US [44]. While biopsy is the gold standard for liver steatosis, several studies have demonstrated that MRS has excellent correlation with the liver fat content from the histopathologic assessment [7,61,89,90]. Interestingly, MRS also shows a better correlation with the actual liver fat content compared to the steatosis assessment performed by histopathologists [78,85,91]. MRS also has high reproducibility across field strength [92] with high inter- (mean ICCs = 0.990) and intrareproducibility (mean ICCs = 0.995) [24], and a low standard deviation of repeated measurement of less than 1% [85,89]. This therefore suggests that MRS continues to be the noninvasive reference standard of choice in both a research and clinical setting.

## 5. Application of MRS for Liver Fat Quantification

MRS has been used in several types of studies to evaluate liver fat due to its noninvasive, sensitive, and accurate nature, especially as a reference standard of choice as mentioned in the previous section. An overview of additional possible applications and previous MRS works for liver fat quantification will be discussed in the following sections.

### 5.1. Evaluation of Diffuse Liver Fat Disposition

MRS has been used in clinical trials to investigate liver steatosis grading. Previous studies have applied MRS to investigate liver steatosis grading in a large group of subjects [77,93]. This illustrates the feasibility of MRS for hepatic steatosis grading in the general population. Due to the increasing prevalence of obesity worldwide, MRS has been used to assess liver fat in obese patients, type 2 diabetes (T2D) patients, and patients at risk of fatty liver [75,93,94,95]. Furthermore, MRS has been used to evaluate liver health assessment recommendations for T2D patients at risk of liver steatosis [96]. MRS is extremely valuable for liver fat content assessment in adolescents and sensitive populations requiring timely intervention. In a study of 105 mother–infant pairs, abdominal adiposity and MRS-measured liver fat in infants was associated with maternal BMI [97]. A previous study demonstrated that a significant increase in liver fat was found in healthy young adults with high BMI compared to controls [75].

Moreover, MRS can estimate the subspecies of liver fat from MRS from the lipid subspecies index using an equation based on the oil spectra model [98,99,100]. It has been suggested that the degree of saturation of liver lipids may be associated with liver fat accumulation on hepatocellular damage and disease progression [99,101]. Each peak of lipid spectra reflects the different chemical positions within the triglyceride molecule, including unsaturated, saturated, monounsaturated, and polyunsaturated fatty acids [102]. The fatty acid composition quantification obtained from MRS has also shown good agreement with other MRI-based methods [103,104]. Recent research used MRS to investigate lipid composition and demonstrated that NAFLD patients showed a significantly increased saturation fatty acid index and significantly decreased unsaturation index [99]. The MRS-assessed saturated fatty acid fraction in the liver is associated with de novo lipogenesis and is higher in NAFLD and T2D patients than in patients without these conditions [105]. In another study, MRS was used in a cohort of suspected and known NAFLD participants and demonstrated that liver fat becomes more saturated as FF is elevated [106].

Several studies have demonstrated that MRS can evaluate the efficiency of therapeutic intervention [107]. MRS was previously used in a clinical trial of drugs for NAFLD and was able to evaluate the reduction in liver fat content in a dose-dependent manner [108,109]. In a double-blind study of NAFLD patients, MRS-assessed liver fat showed no alteration from symbiotic treatment while reducing fecal dysbiosis [110]. Additionally, MRS can also be used to assess the effect of dietary and lifestyle changes on liver fat content. A previous MRS study demonstrated that short-term exercise improved liver lipid saturation, insulin sensitivity, and oxidative stress in individuals with known NAFLD [100]. Another study in overweight participants showed that an excessive saturated fat diet increased insulin resistance and MRS-measured liver fat accumulation more than an unsaturated diet [111]. Additionally, MRS is regarded as an accurate noninvasive tool for liver fat quantification in NASH, the more severe form of NALD [69,112]. Compared to other imaging modalities, MRS measurement is not impeded by obesity, ascites, or inflammation, and is extensively used in NASH pharmacotherapy trials to investigate the fat content alteration [113,114]. Interestingly, MRS has demonstrated the ability to predict steatohepatitis with 100% sensitivity and 89% specificity [90] and is suggested to be more reliable than histopathology [89,115].

MRS has also been used to study liver-related diseases that have fat accumulation within hepatocytes, such as human immunodeficiency virus (HIV), hepatitis C virus (HCV), excessive alcohol consumption, and hepatotoxic effects from chemotherapeutic agents or antiretroviral therapy [21,116,117]. It has been suggested that liver fat accumulation in HCV infection is influenced by both host and viral factors [118]. Liver steatosis in HCV infection is associated with an increased risk of liver fibrosis, accelerated liver necroinflammatory activity, and a lower response rate to antiviral therapy [118,119,120]. Therefore, liver fat MRS could assess pathology progression and response to therapeutic intervention. In a retrospective study, chronic HCV patients who underwent MRS showed liver fat reduction after treatment with direct-acting antiviral therapies [121]. Previous liver fat assessed by MRS showed an increased prevalence of steatosis in patients with HCV genotype 3 [117]. On the other hand, another study showed that patients coinfected with HIV/HCV and HCV monoinfection had reduced liver steatosis, suggesting that infections with HCV genotypes other than 3 may prevent liver fat accumulation [122].

Due to the high accuracy and sensitivity of MRS, it has also been used in liver fat assessment for liver transplants. Hepatic steatosis not only influences the outcome of translation but also increases the complication risk of both participants and donors [123]. It is recommended that donor livers have less than 5% liver fat [21]. A high steatosis level of the donor liver increases the risk of recipient hepatic dysfunction and renal failure [124,125].

### 5.2. Cirrhosis

MRS is a promising tool for the evaluation of liver cirrhosis. Chronic hepatitis can be classified into stages based on the level of fibrosis and necroinflammatory activity insults [126]. Elevated cirrhosis is a risk factor for developing hepatocellular carcinoma (HCC) [127]. Therefore, a correct diagnosis and monitoring of cirrhosis is clinically valuable. MRS has previously been used to investigate alterations in lipid and choline levels in liver cirrhosis patients [128]. A study of chronic hepatitis patients showed that the metabolite-to-lipids ratio increased with the chronic hepatitis stage [129]. This result may be explained by the true increase in metabolite concentrations or the decrease in liver lipid signals [64,129]. The increases in iron disposition within the liver might lead to magnetic field inhomogeneity and thus decrease spectrum resolution [130], potentially introducing errors in metabolite quantification. Careful analysis and interpretation of MRS spectra for liver cirrhosis is therefore needed.

### 5.3. Evaluation of Focal Liver Fat Disposition

In addition to diffuse liver fat assessment, MRS has also been used to investigate metabolite alterations in focal hepatic lesions such as benign lesions and malignancies. In addition to lipids, liver spectra can also be used to investigate choline-containing compounds (i.e., choline, phosphoethanolamine, and phosphocholine). These choline-containing compound peaks cannot be resolved in the low-field MR system and appear as a single peak at 3.2 ppm. The alteration of choline-containing compounds has been hypothesized to be associated with elevated cell membrane turnover and cell proliferation [131]. Abnormal choline-containing compounds are suggested to be associated with carcinogenesis [132]. Previous work on in vitro MRS in liver biopsies demonstrated elevated phosphomonoesters (i.e., phosphothanolamine and phosphocholine) and reduced phosphodiesters (i.e., glycerophosphocholine and glycerophosphoetganoalamine) compared to healthy liver tissue [133]. Several studies have demonstrated that MRS is able to discriminate HCC lesions from cirrhosis and healthy liver [128,134]. One study showed the choline-to-lipids ratio and suggested that the combination of both MRI- and MRS-based imaging may improve sensitivity and specificity for discriminating between benign and malignant lesions [134]. Another study also showed good efficiency (ROC curve = 0.97) in using choline-containing peaks for malignant liver tumor discrimination [135]. In one study, the HCC liver showed higher choline-containing compounds and a higher overall signal peak from overlapping lactate and triglyceride than the cirrhotic liver and cirrhotic liver with HCC [136].

However, there are discrepancies between MRS liver studies for choline-containing compounds in focal hepatic lesions. It was previously demonstrated that choline-containing compounds could be substantially high in the livers of healthy young adults. However, the overall choline-containing compounds are higher in hepatic tumors [137]. In another study of 33 hepatic lesions following transcatheter arterial chemoembolization, the results showed no significant difference in the choline-containing compound-to-lipid ratio observed between normal livers and malignant tumors [134]. One possible explanation is that choline-containing compounds vary between tumors and the degree of necrosis. This different metabolite concentration between viable and necrotic areas dilutes the metabolite alteration seen in viable tumor cells. Another limitation is that MRS requires relatively large voxels, and it is possible that signals outside the lesion could contaminate the acquired spectra. Additionally, these lesions exhibit a low SNR even on high-field MR (>3 Tesla). While the breath-hold approach for MRS acquisition potentially improves the SNR and thus spectral resolution, patients with pathologies may not tolerate this approach well. With higher-field MR, an improved MRS acquisition technique and careful study design could potentially improve the feasibility of MRS for focal hepatic lesions.

MRS has also been used in other studies, such as MRS of the gallbladder, to study the bile component and metastasis of adenocarcinoma of the liver [138,139]. The improvement of higher-field MR systems and acquisition techniques leads to many possible clinical applications of MRS. With increased SNR, MRS might be used to study the liver in a smaller area and with high spectral resolution, thus improving the ability to resolve metabolite features within the tissue of interest. The evidence suggests that MRS is a powerful tool for the noninvasive assessment of liver fat content that can be used in both research and clinical settings. However, performing MRS requires extensive resources, and thus, MRS remains mainly a research modality despite its potential for accurate liver fat quantification in clinical applications.

## 6. Possible Confounders and Limitation of Liver MRS

Several confounders could affect the accuracy of liver fat measurement from MRS. However, this can be avoided by careful design of MRS acquisition and correction of these confounding factors. While MRS spectroscopy is the most direct measurement of liver fat signals, it is affected by T1 bias and T2 relaxation effects, which lead to errors in liver fat fraction measurements [140]. T1 bias occurs from the difference in T1 relaxation times, which leads to relative amplification of the fat signal [76,141]. The T2 relaxation effect results in signal loss with increasing echo time (TE). Both the PRESS and STEAM methods require a delay between TE, therefore enabling spin-spin relaxation and decreasing the signal [142]. It is also commonly known that T2* relaxation is amplified in the presence of iron in the liver [140,143]. Therefore, correction for T1 and T2 relaxation effects is needed for accurate metabolite quantification. A long repetition time (TR) of four to five times the T1 relaxation time (>3000 ms) may be used to minimize the T1 relaxation effect [71]. Multi-echo MRS has also been used to obtain T2 relaxation times for T2 correction using an exponential least-squares algorithm [144].

One of the confounders is motion artifacts. Motion artifacts in liver MRS can arise from several sources, including gross movement, respiration, and cardiac pulsation. Rhythmic motion, such as respiratory and cardiac motion, often leads to phase and frequency shifts, spectrum line broadening, and a reduced degree of water suppression [71]. Additionally, gross movement could lead to voxel misregistration and the contamination of signals outside the voxel. Several techniques have successfully reduced the effect of motion on MRS spectra, including respiratory gating, the navigator pulse technique, and signal averaging. With the improvement of the MR system, MRS spectra can be successfully obtained in a single breath hold [47]. However, this method requires subject cooperation and might not be suitable for all populations.

Another limitation is that the MRS technique for liver fat quantification is complex, expensive, and not widely available. MRS of liver fat requires an MR machine with a homophonous magnetic field with MRS capability, a complex analysis method, a specialized software package, and user expertise to carry out the measurement. Therefore, MRS is mostly used in research and clinical trials rather than clinical settings. In addition, MRS is sampled from only a small area of the liver parenchyma. Therefore, MRS liver FF does not reflect the lipid content of the whole liver [31].

Despite these limitations of MRS, continuous effort is being made towards technical refinement and standardized MRS for translation into the clinical setting. The developments in the MR instrument and software have made the MRS sequence a rather standard addition with a clinical scanner. The improvements of the MR system have led to the reduction of MRS scan time, and the possibility of data acquisition and analysis remote controlled by skilled specialists improves its feasibility in a clinical setting. Additionally, efforts have been made towards automate- or semi-automate data analysis from both the commercially and freely available software mentioned in this review that enable accurate and consistent liver fat MRS measurement [145,146,147]. These improvements, while providing accurate liver fat quantification, therefore propose liver MRS as a time and cost-effective addition to the routine scan.

## 7. Conclusions

Liver steatosis is becoming a worldwide health concern as an independent risk factor for NASH and liver-related morbidity and mortality. NAFLD is also recognized as the hepatic manifestation of metabolic syndrome. The growing prevalence of metabolic syndrome worldwide has led to interest in using noninvasive techniques to investigate hepatic fat.

MRS has been shown to be one of the most precise and accurate techniques for liver fat assessment, and it provides a straightforward quantitative measurement of liver steatosis. Moreover, MRS has the potential to become an alternative substitution for liver biopsy and thus avoid complications such as bleeding and infection. Given the excellent sensitivity of MRS, it also plays an important role in the initial detection of liver fat content and monitoring the response to treatment. While the application of MRS was previously limited to clinical trials and research settings due to its requirement of user expertise, the continued refinement and validation of instrument and acquisition technique now lead to the possibility to incorporate its robust application into clinical routine.

## Figures and Tables

**Table 2:** Detectable metabolite peaks from the liver MR spectrum.

Peak	Chemical Shift (ppm)	Type	Hydrogen Atom Position (Bold)
A	0.9	Methyl	-CH_2_-C**H_3_**
B	1.3	Methylene	-(C**H_2_**)*n*-
C	1.59	β-Carboxyl	-C**H_2_**-CH_2_-COO
D	2.1	α-olefinic	-C**H_2_**-CH=CH-
E	2.25	α-Carboxyl	-CH_2_-C**H_2_**-COO
F	2.75	Diacyl	-CH=CH-C**H_2_**-CH=CH-
-	4.7	Water	**H_2_**O

## Appendix A

### Software Packages for Liver Fat Quantification

Only software packages commonly used for liver fat quantification, linear combination of model spectra (LCModel), and Java-based magnetic resonance user interfaces (jMRUIs), are discussed in this review.

LCModel is an automated commercial software package for MRS data analysis. LCModel analyze data in the frequency domain as a linear combination of a model in vitro [148]. Rather than using individual peaks as prior knowledge for spectral fitting, LCModel incorporates the whole set of spectral peaks of each metabolite. The basis set of the spectral model is supplied with the software package or can be obtained from the basis set of in vitro metabolite solutions acquired under the same conditions as the in vivo data [147,149]. LCModel therefore provides users with flexibility to some extent. However, LCModel is an automated process with a black-box approach and requires user expertise in the modification of the LCModel basic set.

JMRUI (java version, Magnetic Resonance User Interface) is freeware that analyzes MRS data in the time domain [146]. Available online: http://www.mrui.uab.es/mrui/mruiHomePage.htm (accessed on 6 May 2021). JMRUI is equipped with several toolboxes useful for MRS data postprocessing, such as filtering low-frequency signals, water residual suppression tools, phase and frequency corrections, and eddy current artifact corrections [81,150]. Additionally, jMRUI is equipped with several modes of analysis, such as QUantitation based on QUantum ESTimation (QUEST) and Advanced Method for Accurate Robust and Efficient Spectral Fitting (AMARES) [83,151]. Compared to LCModel, jMRUI provides more support for user interaction with the analysis process with a user-friendly interface. However, user interaction with the preprocessing step might influence spectrum fitting and introduce error in metabolite quantification [152].
